# Traffic as a barrier to walking safely in the United States: Perceived reasons and potential mitigation strategies

**DOI:** 10.1016/j.pmedr.2022.102003

**Published:** 2022-09-27

**Authors:** Graycie W. Soto, Geoffrey P. Whitfield, Bryant J. Webber, John D. Omura, Tiffany J. Chen, Hatidza Zaganjor, Kenneth Rose

**Affiliations:** a Division of Nutrition, Physical Activity, and Obesity, National Center for Chronic Disease Prevention and Health Promotion, Centers for Disease Control and Prevention, Atlanta, GA, United States; b Epidemic Intelligence Service, Centers for Disease Control and Prevention, Atlanta, GA, United States; c McKing Consulting Corporation, Atlanta, GA, United States

**Keywords:** Epidemiology, Built environment, Traffic, Walking, Barrier, Safety countermeasures

## Abstract

Motor vehicle traffic is commonly cited as a barrier to walking, but national level perceptions of traffic characteristics that negatively influence walking and potential traffic mitigation strategies remain unclear. The objectives of this study were to describe perceptions of (1) traffic characteristics that make walking unsafe in the United States and (2) potential mitigation strategies to address these concerns among those who report traffic as a barrier to walking. Data were from *FallStyles*, a nationwide internet panel survey conducted in October 2019 (n = 3,284 adults). Respondents reported if traffic makes walking unsafe where they live; those who answered *yes* were then asked about traffic characteristics that make walking unsafe (number of vehicles, speed of vehicles, distracted or impaired driving, types of vehicles, and other reasons) and potential mitigation strategies (new or improved sidewalks, crosswalks, pedestrian signals, street lighting, things that slow vehicles down, separating the sidewalk from the road, fewer vehicle lanes, and other). Prevalence of responses was assessed overall and by select sociodemographic and geographic characteristics, and by walking status. Nearly 25% of US adults reported that traffic is a barrier to walking where they live. Of these, 79% selected vehicle speed as a contributing traffic characteristic, and 57% indicated new or improved sidewalks as a potential mitigation strategy. These top responses were shared across all sociodemographic, geographic, and walking behavior subgroups. Speed reduction efforts and built environment enhancements such as sidewalks may alleviate pedestrian safety concerns. Promotion campaigns may be needed to bring awareness to such changes.

## Highlights

• Almost a quarter of US adults perceive traffic as a barrier to safe walking.
• Vehicle speed is the leading perceived traffic characteristic of concern.
• Respondents perceive sidewalks as the most favorable strategy to mitigate risk.

## 1 Introduction

Physical activity is one of the most important things people can do for their health (2018 Physical Activity Guidelines Advisory Committee, 2018). To achieve substantial health benefits, adults are encouraged to do at least 150 minutes per week of moderate-intensity aerobic physical activity, 75 minutes per week of vigorous-intensity aerobic physical activity, or an equivalent combination (US Department of Health and Human Services, 2018). Walking is the most common physical activity reported by US adults (Watson et al., 2015) and forms the foundation of *Step it Up! The Surgeon General’s Call to Action to Promote Walking and Walkable Communities* (US Department of Health and Human Services, 2015).

Motor vehicle traffic is a noted barrier to walking in the United States (Whitfield et al., 2018b). Measurable traffic conditions that pose a risk to pedestrians, such as vehicle speed and volume, have long been established in the transportation and injury prevention literature (Leaf and Preusser, 1999, Stoker et al., 2015, Wazana et al., 1997). Conversely, less is known about people’s perceptions of traffic as a barrier to walking. Previous studies have attempted to quantify the association between traffic and physical activity by computing a composite score that reflects people’s general perceived safety from traffic (Bracy et al., 2014, Saelens et al., 2003). Others have investigated perceptions of specific elements of traffic, such as vehicle speed and volume, that may make it a barrier to walking. Many such studies have been conducted outside the United States (Anciaes et al., 2019, Cleland et al., 2008, Ferrari et al., 2020) or in a small number of geographic study locations (Lee et al., 2021, McGinn et al., 2007, Stewart et al., 2016), and are therefore less informative for the broader US population.

There is also ample evidence about engineering and design strategies to increase pedestrian safety from traffic (Campbell et al., 2004, Harkey and Zegeer, 2004, Retting et al., 2003, Zegeer and Bushell, 2012). Traffic engineering countermeasures to improve pedestrian safety from motor vehicles can be broadly described in 3 categories: managing vehicle speeds, separating pedestrians and vehicles, and increasing pedestrian visibility (Retting et al., 2003). Many studies have evaluated the effect of interventions that implement these countermeasures in specific cities in the United States (Chen et al., 2013, Redmon, 2011). In 2008, the Federal Highway Administration launched the Proven Safety Countermeasures Initiative, a resource for transportation agencies to access information on data-driven strategies to reduce roadway fatalities (Albee & Bobitz, 2021). In contrast to the substantial literature on efficacy, few studies have described the general public’s preferences for these various mitigation strategies, with existing studies restricted to one geographic area (Emo et al., 2011) or outside the United States (Aceves-González et al., 2020, Anciaes et al., 2017).

It is important to understand perceptions of the built environment in addition to objective measures because perceptions may affect behavior regardless of what is objectively measured in the environment (Loukaitou-Sideris, 2006). Even environments with few objectively measured risks to pedestrian safety may disincentivize walking if viewed by pedestrians as subjectively unsafe. A built environment characteristic that is desirable to someone with a high tolerance for risk could be perceived as a threat to someone with a low tolerance for risk (Bjornstrom & Ralston, 2014), such as a narrow sidewalk that is present but not separated from high-speed traffic by street trees, street furniture, or other buffer elements (National Association of City Transportation Officials, 2013). Perceptions of safety may be influenced by individual factors, including prior experiences, sociodemographic characteristics, and contextual factors, such as physical or social neighborhood incivilities (Loukaitou-Sideris, 2006). The built environment may also be perceived more favorably by people who walk in their neighborhood and are therefore exposed more frequently to the environment (Herbolsheimer et al., 2020). This may indicate that people who walk for transportation out of necessity have different perceptions of the environment than people who walk for leisure. Because the environment is likely experienced differently by all people, it is important to stratify perceptions of the built environment by subgroups.

Evidence about public perceptions of traffic and built environment safety interventions could help to direct efforts to communities most in need and tailor interventions to specific subgroups and neighborhood conditions (Loukaitou-Sideris, 2006). Our study aimed to expand upon previous work that has identified traffic as a barrier to walking (Whitfield et al., 2018b) by using national survey data to assess perceptions of multiple characteristics of motor vehicle traffic that might contribute to pedestrian safety concerns and to identify potential safety mitigation strategies. Among respondents who report traffic as a barrier to walking, we sought to describe their perceptions of (1) traffic characteristics that make walking unsafe and (2) potential mitigation strategies that would diminish this safety concern.

## 2 Methods

### 2.1 Survey and analytic sample

Porter Novelli’s *Styles* database is built from a series of online-based surveys via Ipsos’ KnowledgePanel®, a panel representative of the non-institutionalized US population. Panel members are randomly recruited using probability-based sampling by home address. The panel is continuously replenished and maintains approximately 55,000 panelists. The *SpringStyles* survey was sent to 11,012 adult panelists and fielded from 3/27/2019–4/15/2019, with 6,657 panelists completing the survey (response rate = 60.5%). The *FallStyles* survey was fielded from 10/8/2019–10/22/2019 to a random sample of 4,677 adult panelists who previously completed the *SpringStyles* survey, and 3,624 completed the survey (response rate = 77.5%). Respondents received reward points worth approximately $5. Data were weighted to match the 2018 US Current Population Survey proportions for sex, age, household income, race/ethnicity, household size, education, census region, and metro status (US Census Bureau, 2018). We excluded respondents who did not answer questions related to walking (n = 196) or traffic (n = 144), yielding a final analytic sample of 3,284.

### 2.2 Measures

#### 2.2.1 Traffic

Respondents were asked, “Where you live, does traffic make it unsafe for you to walk?” Respondents could choose *yes*, *no*, or *don’t know*. Those who answered *yes* were classified as perceiving traffic as a barrier to walking. Those who identified traffic as a barrier were then asked about traffic characteristics causing unsafe walking conditions and related potential mitigation strategies, henceforth referred to as “mitigation strategies.” For traffic characteristics, respondents were asked, “Where you live, which of the following are reasons traffic make it unsafe for you to walk?” Respondents could select all that applied, and response options included, “Number of vehicles,” “Speed of vehicles,” “Distracted or impaired driving,” “Types of vehicles (e.g., large trucks),” and “Other reasons.” For mitigation strategies, respondents were asked, “Where you live, which of the following would make traffic less of a barrier for you to safely walk?” Respondents could select all that applied, and response options included, “New or improved sidewalks,” “Crosswalks,” “Pedestrian signals,” “Street lighting,” “Things that slow vehicles down (e.g., speed humps, traffic circles, curb extensions),” “Separating the sidewalk from the road,” “Fewer vehicle lanes,” and “Other.” The prevalence of “other reasons” traffic makes it unsafe to walk and “other” mitigation strategies was reported as a footnote but not interpreted due to lack of specificity.

#### 2.2.2 Walking behavior

To assess walking behavior, respondents were asked how many days they walked in the past week, and how many minutes per day, for transportation (“to and from work, to do errands, or to go from place to place”) and for leisure (“for fun, relaxation, or exercise”). Respondents were classified as transportation walkers if they reported any amount of walking for transportation and as leisure walkers if they reported any amount of walking for leisure. Classifications were not mutually exclusive.

#### 2.2.3 Demographic characteristics

Respondent characteristics included age (18–34, 35–49, 50–64, and ≥ 65 years), sex, race/ethnicity (White, non-Hispanic; Black, non-Hispanic; Hispanic; Other and multiracial, non-Hispanic), education level (high school or less, some college, Bachelor’s degree or higher), household income (<$50,000, $50,000–$99,999, and ≥$100,000), region (Northeast, Midwest, South, West), and metropolitan status (non-metro or metro) (US Census Bureau, 2021).

### 2.3 Statistical analysis

Weighted prevalence and 95% confidence intervals were calculated overall and by respondent characteristics (age, sex, race/ethnicity, education level, household income, region, metropolitan status, transportation walking, and leisure walking) for the following: (1) perceptions of traffic as a barrier to walking; (2) traffic characteristics that make walking unsafe among those reporting traffic as a barrier to walking; and (3) potential mitigation strategies among those reporting traffic as a barrier to walking. Significant differences were identified using adjusted Wald tests and pairwise t tests with Bonferroni correction; trends were tested using orthogonal polynomial contrasts. Significance level was *P* < 0.05. Variables with a relative standard error greater than 30% were suppressed. All analyses were conducted in SAS (v 9.4) using survey procedures to account for weighting. Descriptive characteristics were calculated with and without sample weights. Institutional review board approval was not required because no personal identifiers were included in the data file. The study was conducted according to applicable federal law and Centers for Disease Control and Prevention (CDC) policy.

## 3 Results

### 3.1 Sample characteristics

The majority of the unweighted sample was male, aged ≥ 50 years, non-Hispanic White, had at least some college education, had a household income of ≥$50,000, and lived in a metro area (Table 1).

**Table 1:** Sample Characteristics and Prevalence of Reporting Traffic as a Barrier to Walking, 2019 FallStyles.

	Sample Characteristics			Reported Traffic as a Barrier to Walking	
	N	Unweighted %	Weighted %	Weighted %	95 % CI
Total	3284	–	–	23.7	(21.9, 25.5)
Age (years)					
18–34	530	16.1	28.1	28.5^L^	(24.1, 32.8)
35–49	702	21.4	24.5	25.9	(22.3, 29.4)
50–64	1090	33.2	26.7	20.9	(18.3, 23.5)
65+	962	29.3	20.7	18.3	(15.5, 21.1)
Sex					
Male	1737	52.9	48.8	20.6^x^	(18.3, 23.0)
Female	1547	47.1	51.2	26.6^y^	(24.1, 29.2)
Race/ethnicity					
White, NH	2431	74.0	64.9	22.3	(20.4, 24.3)
Black, NH	261	7.9	11.1	21.8	(16.0, 27.6)
Hispanic	333	10.1	15.7	30.4	(24.9, 36.0)
Other and multiracial, NH	259	7.9	8.3	24.2	(17.9, 30.5)
Education level					
HS or less	1041	31.7	37.6	25.3	(22.1, 28.5)
Some college	960	29.2	28.1	24.7	(21.5, 28.0)
Bachelor's degree or higher	1283	39.1	34.3	21.1	(18.6, 23.7)
Income					
Less than $50,000	965	29.4	30.9	30.9^L^	(27.3, 34.5)
$50,000–$99,999	1021	31.1	32.1	23.6	(20.5, 26.8)
$100,000 or more	1298	39.5	37.0	17.8	(15.4, 20.1)
Region					
Northeast	613	18.7	18.1	24.4x, y	(20.2, 28.6)
Midwest	749	22.8	20.6	18.3^x^	(14.9, 21.8)
South	1169	35.6	37.2	28.0^y^	(25.0, 31.1)
West	753	22.9	24.1	21.1^x^	(17.6, 24.5)
MSA status					
Non-metro	443	13.5	13.1	20.2	(15.8, 24.5)
Metro	2841	86.5	86.9	24.2	(22.3, 26.1)
Transportation walking					
No	2402	73.1	71.3	23.4	(21.3, 25.4)
Yes	882	26.9	28.7	24.6	(21.1, 28.1)
Leisure walking					
No	1197	36.4	38.8	26.3^x^	(23.3, 29.2)
Yes	2087	63.6	61.2	22.1^y^	(19.9, 24.2)

Abbreviations: CI = confidence interval; MSA = metropolitan statistical area; NH = non-Hispanic and HS = high school.

L For ordinal variables, linear trends across the categories were assessed; superscript L indicates a significant linear trend within demographic subgroup (*p* < 0.05).

x,y Indicate significant differences within demographic subgroups; values with different letters are significantly different (Bonferroni corrected *p* < 0.05); values that do not have a superscript are not significantly different.

### 3.2 Perceptions of traffic as a barrier to walking

Of all respondents, 23.7% reported that, where they live, motor vehicle traffic was a barrier to walking (Table 1). Prevalence was greater among females than males, among those living in the South than those living in the Midwest and West, and among those who did not walk for leisure than among leisure walkers. Prevalence of perceptions of traffic as a barrier to walking decreased linearly by age and income level.

### 3.3 Perceived traffic characteristics that make walking unsafe

Among those who reported traffic as a barrier to walking, vehicle speed was the leading concern overall and across all subgroups; speed was selected by ≥ 75% of respondents in nearly all sociodemographic and geographic subgroups (Table 2). Number of vehicles and distracted or impaired driving were the second and third leading concerns, respectively. Respondents who lived in metro areas, compared to those in non-metro areas, more frequently identified distracted or impaired driving as a concern. Vehicle type was the least common concern. No statistically significant sociodemographic or geographic subgroup differences were noted for speed, number, or types of vehicles.

**Table 2:** Prevalence of Perceived Traffic Characteristics That Make Walking Unsafe Among Those Who Report Traffic as a Barrier to Walking, 2019 FallStyles (N = 710).

	Speed of Vehicles % (95 % CI)	Number of Vehicles % (95 % CI)	Distracted or Impaired Driving % (95 % CI)	Types of Vehicles % (95 % CI)
Total	78.8 (75.3, 82.3)	63.1 (58.9, 67.2)	38.8 (34.6, 42.9)	31.9 (27.9, 35.9)
Age (years)				
18–34	83.9 (77.1, 90.6)	66.7 (58.1, 75.4)	37.3 (28.9, 45.8)	35.3 (26.7, 43.9)
35–49	76.2 (69.3, 83.2)	60.5 (52.6, 68.4)	36.7 (29.1, 44.2)	29.3 (22.3, 36.4)
50–64	78.5 (72.7, 84.2)	65.8 (59.2, 72.4)	44.6 (37.7, 51.6)	31.9 (25.4, 38.3)
65+	72.8 (64.8, 80.8)	55.6 (46.9, 64.3)	36.6 (28.1, 45.1)	29.3 (21.0, 37.7)
Sex				
Male	76.3 (70.8, 81.8)	61.9 (55.5, 68.4)	42.6 (36.1, 49.0)	28.8 (23.0, 34.7)
Female	80.6 (76.1, 85.2)	63.9 (58.5, 69.4)	35.9 (30.6, 41.3)	34.2 (28.8, 39.7)
Race/ethnicity				
White, non-Hispanic	80.0 (76.1, 83.9)	63.0 (58.2, 67.9)	37.7 (32.9, 42.5)	32.3 (27.5, 37.1)
Black, non-Hispanic	78.8 (66.0, 91.6)	62.2 (47.6, 76.8)	31.0 (17.6, 44.4)	30.5 (16.2, 44.8)
Hispanic	79.3 (70.2, 88.4)	61.9 (51.0, 72.7)	41.1 (30.4, 51.9)	31.3 (21.4, 41.2)
Other and multiracial, non-Hispanic	68.6 (54.7, 82.5)	67.3 (52.7, 82.0)	49.9 (34.8, 65.0)	32.4 (18.6, 46.3)
Education level				
High school or less	78.5 (72.4, 84.5)	59.4 (52.0, 66.7)	34.5 (27.5, 41.5)	32.2 (25.2, 39.2)
Some college	78.3 (71.9, 84.7)	67.6 (60.7, 74.6)	40.7 (33.2, 48.1)	32.0 (24.9, 39.0)
Bachelor's degree or higher	79.7 (74.1, 85.2)	63.6 (56.9, 70.3)	42.4 (35.7, 49.2)	31.5 (25.0, 38.0)
Income				
Less than $50,000	79.4 (73.5, 85.3)	64.2 (57.5, 71.0)	36.9 (30.2, 43.5)	36.9 (30.1, 43.7)
$50,000–$99,999	79.7 (73.6, 85.9)	61.0 (53.3, 68.7)	36.4 (29.0, 43.8)	30.3 (23.3, 37.4)
$100,000 or more	76.8 (70.8, 82.8)	63.8 (56.8, 70.7)	44.2 (37.0, 51.5)	26.6 (19.9, 33.2)
Region				
Northeast	79.6 (72.0, 87.3)	63.8 (53.9, 73.7)	38.0 (28.6, 47.5)	35.8 (26.0, 45.7)
Midwest	77.3 (68.2, 86.4)	63.7 (53.4, 73.9)	33.4 (23.7, 43.1)	25.5 (16.8, 34.2)
South	80.4 (75.5, 85.4)	61.8 (55.6, 68.0)	36.0 (29.8, 42.1)	32.7 (26.6, 38.8)
West	75.8 (67.3, 84.3)	64.7 (55.6, 73.7)	49.1 (39.8, 58.3)	31.8 (23.2, 40.3)
MSA status				
Non-metro	82.7 (73.7, 91.7)	58.3 (46.8, 69.9)	25.1 (15.5, 34.6)^x^	37.8 (25.8, 49.8)
Metro	78.3 (74.5, 82.1)	63.7 (59.2, 68.1)	40.5 (36.0, 44.9)^y^	31.2 (26.9, 35.5)

Abbreviations: CI = confidence interval and MSA = metropolitan statistical area.

The prevalence of reporting other reasons overall was 21.6%.

x,y Indicate significant differences within demographic subgroups; values with different letters are significantly different (Bonferroni corrected *p* < 0.05); values that do not have a superscript are not significantly different.

**Fig. 1:**
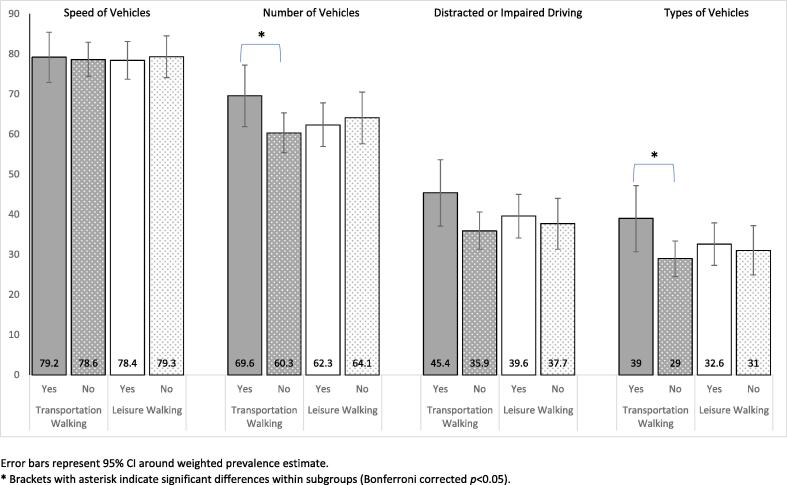
Prevalence of perceived traffic characteristics that make walking unsafe among those who report traffic as a barrier to walking, by participation in transportation and leisure walking (n = 710).

Transportation walkers were more likely than those who did not walk for transportation to report walking safety concerns due to number and type of vehicles. No significant associations were found between leisure walking status and perceived traffic characteristics that make walking unsafe (Fig. 1).

### 3.4 Mitigation strategies

Among those who reported traffic as a barrier to walking, new or improved sidewalks was the most commonly selected mitigation strategy overall and across subgroups (Table 3). The second most commonly selected mitigation strategy overall was separating the sidewalk from the road. For both of these strategies, prevalence was similar across categories of each sociodemographic and geographic characteristic, and by categories of walking behavior. Some mitigation strategies exhibited variability by race/ethnicity. For example, Hispanic respondents were more likely than non-Hispanic White respondents to select strategies to slow vehicles and to improve pedestrian signals. Additional subgroup differences for some potential mitigation strategies were observed by metropolitan status, transportation walking, and leisure walking. There was a linear association between crosswalks and age: younger respondents selected crosswalks more frequently than older respondents. Only 10% of adults who reported traffic as a barrier to walking selected fewer vehicle lanes as a potential mitigation strategy.

**Table 3:** Prevalence of Potential Mitigation Strategies Among Those Who Report Traffic as a Barrier to Walking, 2019 FallStyles (N = 710).

	New or Improved Sidewalks % (95 % CI)	Separating the Sidewalk From the Road % (95 % CI)	Things That Slow Vehicles Down % (95 % CI)	Crosswalks % (95 % CI)	Street Lighting % (95 % CI)	Pedestrian Signals % (95 % CI)	Fewer Vehicle Lanes % (95 % CI)
Total	56.8 (52.5, 61.0)	45.1 (40.8, 49.3)	38.5 (34.4, 42.7)	34.3 (30.1, 38.4)	30.5 (26.6, 34.5)	27.6 (23.8, 31.5)	10.0 (7.4, 12.7)
Age (years)							
18–34	55.9 (47.0, 64.8)	47.0 (38.0, 55.9)	37.6 (29.0, 46.3)	44.6 (35.7, 53.5)^L^	31.5 (23.3, 39.6)	30.5 (22.4, 38.6)	10.6 (5.3, 15.9)
35–49	57.9 (50.0, 65.8)	40.7 (32.8, 48.5)	38.8 (30.9, 46.7)	32.1 (24.5, 39.6)	35.2 (27.5, 42.8)	32.4 (24.7, 40.0)	11.7 (6.3, 17.1)
50–64	55.1 (48.2, 62.1)	50.4 (43.5, 57.4)	38.2 (31.3, 45.0)	29.0 (22.6, 35.5)	27.1 (20.8, 33.4)	20.0 (14.4, 25.6)	9.1 (4.7, 13.5)
65+	59.1 (50.4, 67.7)	40.7 (32.1, 49.2)	40.6 (31.9, 49.4)	24.0 (16.3, 31.6)	26.0 (18.2, 33.9)	25.0 (16.8, 33.1)	–
Sex							
Male	56.2 (49.7, 62.7)	44.6 (38.1, 51.1)	36.4 (30.1, 42.8)	32.0 (25.9, 38.2)	30.5 (24.4, 36.6)	27.7 (21.8, 33.6)	7.8 (4.2, 11.4)
Female	57.2 (51.6, 62.8)	45.5 (39.9, 51.1)	40.1 (34.5, 45.7)	35.9 (30.4, 41.5)	30.6 (25.4, 35.8)	27.6 (22.4, 32.8)	11.7 (7.9, 15.5)
Race/ethnicity							
White, NH	57.9 (52.9, 62.9)	50.1 (45.1, 55.1)	33.4 (28.7, 38.0)^x^	29.7 (25.1, 34.3)	27.8 (23.3, 32.2)	22.0 (17.9, 26.0)^x^	7.3 (4.5, 10.0)
Black, NH	51.8 (36.6, 66.9)	31.7 (18.3, 45.1)	39.3 (24.2, 54.3)x, y	44.0 (28.5, 59.5)	27.6 (13.9, 41.4)	24.5 (11.2, 37.8)x, y	–
Hispanic	58.5 (47.7, 69.3)	38.6 (28.3, 49.0)	49.8 (38.9, 60.7)^y^	41.1 (30.3, 51.9)	36.3 (25.6, 46.9)	38.3 (27.5, 49.1)^y^	–
Other and multiracial, NH	50.2 (35.1, 65.2)	40.3 (25.2, 55.5)	48.2 (33.2, 63.3)x, y	39.7 (24.6, 54.8)	40.4 (25.8, 55.0)	47.3 (32.3, 62.3)^y^	–
Education level							
HS or less	54.1 (46.7, 61.5)	39.7 (32.5, 46.9)	42.1 (34.8, 49.4)	32.8 (25.6, 39.9)	29.7 (22.8, 36.5)	26.2 (19.3, 33.0)	10.8 (6.1, 15.5)
Some college	58.8 (51.3, 66.4)	47.6 (40.0, 55.3)	35.0 (27.6, 42.4)	32.3 (24.9, 39.6)	25.1 (18.4, 31.8)	27.7 (20.8, 34.5)	9.3 (4.4, 14.3)
Bachelor's degree or higher	58.4 (51.6, 65.1)	49.8 (42.9, 56.7)	37.3 (30.7, 44.0)	38.2 (31.4, 45.0)	37.0 (30.3, 43.6)	29.5 (23.3, 35.8)	9.7 (5.7, 13.7)
Income							
Less than $50,000	53.6 (46.5, 60.7)	41.1 (34.2, 48.1)	41.8 (34.7, 48.8)	34.2 (27.2, 41.2)	30.8 (24.2, 37.5)	29.5 (22.8, 36.1)	9.6 (5.4, 13.9)
$50,000–$99,999	56.5 (48.9, 64.1)	45.3 (37.6, 53.0)	36.1 (28.7, 43.6)	34.0 (26.7, 41.4)	25.8 (19.0, 32.6)	26.8 (19.9, 33.7)	11.0 (6.1, 16.0)
$100,000 or more	61.7 (54.7, 68.7)	50.6 (43.2, 57.9)	36.6 (29.7, 43.6)	34.7 (27.7, 41.7)	35.6 (28.6, 42.6)	25.9 (19.6, 32.2)	9.5 (4.7, 14.3)
Region							
Northeast	56.5 (46.6, 66.5)	43.9 (33.8, 54.1)	39.6 (29.8, 49.4)	37.7 (27.8, 47.5)	33.1 (23.7, 42.5)	20.9 (13.2, 28.6)	–
Midwest	50.7 (40.0, 61.3)	46.1 (35.6, 56.5)	33.2 (23.6, 42.9)	33.6 (23.2, 43.9)	24.4 (15.6, 33.1)	25.3 (16.2, 34.3)	–
South	62.0 (55.8, 68.2)	47.7 (41.3, 54.1)	37.5 (31.2, 43.8)	32.9 (26.8, 39.1)	29.0 (23.1, 34.8)	26.6 (20.7, 32.6)	10.5 (6.4, 14.6)
West	50.8 (41.5, 60.1)	40.0 (31.1, 49.0)	43.7 (34.5, 53.0)	34.6 (25.4, 43.8)	36.2 (27.0, 45.3)	37.3 (28.1, 46.5)	11.5 (5.3, 17.7)
MSA status							
Non-metro	55.4 (43.4, 67.5)	39.6 (27.7, 51.4)	25.0 (13.8, 36.2)^x^	19.8 (10.1, 29.5)^x^	20.3 (10.7, 29.9)^x^	–	–
Metro	56.9 (52.4, 61.5)	45.8 (41.2, 50.3)	40.2 (35.8, 44.7)^y^	36.1 (31.6, 40.6)^y^	31.8 (27.6, 36.1)^y^	29.3 (25.1, 33.5)	10.7 (7.8, 13.6)
Transportation walking							
No	57.3 (52.3, 62.2)	47.2 (42.2, 52.1)	35.1 (30.4, 39.8)^x^	32.2 (27.4, 37.0)	27.0 (22.7, 31.3)^x^	24.1 (19.9, 28.3)^x^	8.2 (5.4, 11.1)
Yes	55.6 (47.4, 63.8)	40.2 (32.2, 48.2)	46.7 (38.3, 55.0)^y^	39.2 (31.0, 47.4)	38.9 (30.7, 47.2)^y^	36.1 (27.8, 44.3)^y^	14.3 (8.4, 20.2)
Leisure walking							
No	54.4 (47.9, 61.0)	42.7 (36.0, 49.3)	35.6 (29.2, 41.9)	32.4 (26.2, 38.7)	24.8 (19.4, 30.2)^x^	22.3 (16.8, 27.8)^x^	9.5 (5.6, 13.4)
Yes	58.5 (52.9, 64.1)	46.9 (41.4, 52.5)	40.8 (35.3, 46.3)	35.7 (30.1, 41.2)	34.9 (29.4, 40.4)^y^	31.7 (26.4, 37.1)^y^	10.4 (6.8, 14.1)

Abbreviations: CI = confidence interval and MSA = metropolitan statistical area; NH = non-Hispanic and HS = high school.

The prevalence of reporting other mitigation strategies overall was 11.1%.

Dashed line indicates numbers were suppressed due to unstable estimates with relative standard error greater than 30%.

L For ordinal variables, linear trends across the categories were assessed; superscript L indicates a significant linear trend within demographic subgroup (*p* < 0.05).

x,y Indicate significant differences within demographic subgroups; values with different letters are significantly different (Bonferroni corrected *p* < 0.05); values that do not have a superscript are not significantly different.

## 4 Discussion

Nearly 1 in 4 US adults reported traffic as a barrier to walking where they live. Among these adults, vehicle speed was the most commonly perceived traffic characteristic of concern (78.8%), and this was notably consistent across all subgroups. Further, adults who reported traffic as a barrier to walking most often selected mitigation strategies related to sidewalk improvements: nearly 57% selected new or improved sidewalks, and 45% selected separating sidewalks from roads. These findings suggest that speed reduction and improved sidewalk infrastructure may be important interventions for improving perceptions of walkability among US adults.

Our study builds on previous evidence in several important ways. First, other studies on perceptions of traffic as a barrier to walking often relied on a composite score to assess pedestrian safety from traffic, which is helpful but cannot pinpoint the specific traffic characteristics that people perceive as safety concerns while walking. For example, several studies used subscales of the Neighborhood Environment Walkability Scale to measure perceptions of environmental characteristics related to physical activity (Bracy et al., 2014, Carlson et al., 2014, Saelens et al., 2003, Shigematsu et al., 2009). Studies using these subscales have shown mixed associations between physical activity and pedestrian safety from traffic in general and could benefit from more precise measures to inform specific interventions. Second, in contrast to studies that were limited to narrow geographic areas (Carlson et al., 2014, Nehme et al., 2016), our study uses a national sample, which may improve generalizability. Third, our study provides novel information on pedestrian perceptions of potential mitigation strategies—a uniquely actionable addition to what is known about traffic and walking in the United States. Quantifying and stratifying perceptions of potential mitigation strategies provides national data that local communities might use as comparison measures.

Our results regarding perceptions of traffic as a walking barrier largely correspond to a previous study. Analyzing the responses of a representative sample of US adults from the National Health Interview Survey (NHIS), Whitfield and colleagues found that 23.4% of US adults cited traffic as a safety concern related to walking near their home (Whitfield et al., 2018b). This is nearly identical to the prevalence (23.7%) among respondents in our study. Likewise, Whitfield and colleagues also reported a higher prevalence among those with a lower socioeconomic status (assessed by income in our study and education in theirs). The concordance between these studies is encouraging considering the purposefully sampled nature of NHIS.

We observed that speed is consistently perceived as a problem by US adults who report traffic as a barrier to walking. Objective collision research supports vehicle speed as a real danger to pedestrians, meaning these perceptions are well-founded. For example, vehicle speed is directly associated with increased injury severity among pedestrians and higher rates of pedestrian fatalities (Board, 2017, Tefft, 2013). This is especially an issue for arterial roads, which are designed to move high volumes of cars at high speeds but often lack pedestrian friendly infrastructure (McAndrews et al., 2017). Despite making up only 10% of roadways in the United States, non-freeway arterials accounted for more than half of all fatal crashes involving pedestrians in 2019 (Federal Highway Administration, 2021; Governors Highway Safety Association, 2021). Moreover, the problem is worsening. Pedestrian fatalities in the United States increased by 65% from 4,302 in 2010 to 6,516 in 2020 (National Center for Statistics and Analysis., 2021, Stewart, 2022). Speed reduction efforts, especially in areas with high prevalence of pedestrian-related collisions, may mitigate this concerning trend (Federal Highway Administration, 2013).

Preferred mitigation strategies reported by participants in our study were not aligned with current guidance on speed reduction for improved safety. In response to the growing number of all roadway fatalities, the US Department of Transportation recently released the National Roadway Safety Strategy (US Department of Transportation, 2022). One of the strategy’s objectives focuses on promoting safer speeds by re-engineering roads to naturally slow vehicles with design mechanisms that consider the purpose and use of the road and the types of potential road users. One way to reduce vehicle speeds is through lane reduction and reconfiguration (Tan, 2011). Given the near ubiquity of speed as an important factor in our results, it is notable that only 10% of respondents who reported traffic as a barrier to walking selected “fewer vehicle lanes” as a possible mitigation strategy. Since lane reduction is a common strategy used by transportation engineers to reduce vehicle speed, it may indicate that the general public does not consider lane reductions to be an improvement. Collaborative messaging between public health professionals and planners may be beneficial for improving communication about the safety potential for these mitigation strategies and help dispel misconceptions about potential drawbacks. For example, when correctly implemented, lane reductions may not negatively impact traffic flow and congestion (Federal Highway Administration, 2013, Administration, 2016). Even if minor delays are incurred, previous evidence suggests a large majority of adults favor or strongly favor safer street design even if driving is slower (Carlson et al., 2018). It will also be important to bring awareness to built environment modifications through programmatic or advertising campaigns. Additionally, future research could assess the impact of these mitigation strategies on objective walking behavior.

Public perceptions of the need for sidewalks to address pedestrian safety concerns in their community in this analysis are consistent with recommendations in *Step it Up! The Surgeon General’s Call to Action to Promote Walking and Walkable Communities*. This report stresses the importance of walkable community design with basic features such as sidewalks, where walking and rolling is safe and easy for everyone (US Department of Health and Human Services, 2015). Sidewalks are a foundational strategy to increase walkability, but data from 2015 suggest that approximately one-third of adults do not report sidewalks on most streets near their home (Whitfield et al., 2018a). Even when present, improvements to sidewalks may be needed, as sidewalks are most usable when they are maintained and of good quality, free from obstacles and hazards such as cracks, overgrown vegetation, or uneven surfaces (US Department of Health and Human Services, 2015). Separating pedestrians from vehicles by physical barriers such as trees, parklets, and vehicle or bike parking also creates a safe environment for walking (National Association of City Transportation Officials, 2013). However, no consistent evidence on sidewalk conditions or quality exists at the national level. Improved surveillance of sidewalk presence, conditions, and characteristics could help better monitor progress towards national walking goals. Such data could help identify gaps and provide an evidence base for addressing disparities in walking and walkability.

Finally, previous research has shown that built environment correlates of walking differ between transportation and leisure walking (Owen et al., 2004, Saelens and Handy, 2008, Sugiyama et al., 2012, Whitfield et al., 2019). Our study extends this research by examining perceptions of traffic characteristics that make walking unsafe and potential mitigation strategies, stratified by leisure and transportation walking behaviors. Compared to those who did not walk for transportation, transportation walkers were more likely to report number and type of vehicles as concerning characteristics of traffic and to select mitigation strategies that slow vehicles down. These differences were not observed by leisure walking status. These contrasting findings may in part be explained by differing availability of route choices: while leisure walkers may have flexibility to choose routes that avoid high traffic areas, transportation walkers may out of necessity use routes that lead to their destination, irrespective of traffic (Bunds et al., 2019, Weinstein Agrawal et al., 2008). Strategies that combine land use and environmental design with active transportation systems create activity-friendly routes to everyday destinations and are a key component of the CDC’s *Active People, Healthy Nation* initiative (Centers for Disease Control and Prevention, n.d.). The integration of these approaches can create safe walking spaces for all purposes and encourage community participation in physical activities.

### 4.1 Limitations

This study has several limitations. First, respondents were recruited from an internet panel, which may introduce self-selection bias. When compared to the random digit dialing method, however, panel approaches to surveys have generally yielded equivalent results (Fisher and Kane, 2004, Pollard, 2002). Second, the self-reported data may be susceptible to recall and social desirability biases. Third, the first survey question related to traffic is not necessarily specific to motor vehicle traffic. However, the answer choices for the questions related to traffic characteristics that make walking unsafe and mitigation strategies imply vehicular traffic. Fourth, potential mitigation strategies may not have been fully understood. For example, respondents may not have known that reduced vehicle lanes slow traffic. Lastly, the set of potential answers may not capture all perceived characteristics of traffic that make walking unsafe, such as air pollution or noise (Bonaccorsi et al., 2020, Bunds et al., 2019), or all potential mitigation strategies, such as speed limit reduction (Bornioli et al., 2020, Nightingale et al., 2022). Future qualitative research, or more robust quantitative research, may elicit this information.

## 5 Conclusion

Motor vehicle traffic is perceived as a barrier to walking for nearly one-quarter of US adults. Of those who perceive traffic as a barrier, vehicle speed is their leading concern, and over half identified new or improved sidewalks as a potential mitigation strategy. Given the health benefits of walking (US Department of Health and Human Services, 2015) and the importance of walking to promote physical activity participation (US Department of Health and Human Services, 2018, Watson et al., 2015), communities may consider speed reduction and infrastructure supports to provide supportive and safe environments for walking. In addition to changing the built environment, communities may also consider promotion campaigns to bring awareness to such changes.

## Disclaimer

The findings and conclusions in this report are those of the authors and do not necessarily represent the official position of the Centers for Disease Control and Prevention. This research did not receive any specific grant from funding agencies in the public, commercial, or not-for-profit sectors.

## CRediT authorship contribution statement

**Graycie W. Soto:** Conceptualization, Methodology, Software, Formal analysis, Writing – original draft, Writing – review & editing. **Geoffrey P. Whitfield:** Conceptualization, Methodology, Writing – review & editing. **Bryant J. Webber:** Writing – review & editing. **John D. Omura:** Conceptualization, Methodology, Writing – review & editing. **Tiffany J. Chen:** Conceptualization, Methodology, Writing – review & editing. **Hatidza Zaganjor:** Writing – review & editing. **Kenneth Rose:** Writing – review & editing.

## Declaration of Competing Interest

The authors declare that they have no known competing financial interests or personal relationships that could have appeared to influence the work reported in this paper.

## Data Availability

The authors do not have permission to share data.
